# Pelvic Radiation Therapy Induced Vaginal Stenosis: A Review of Current Modalities and Recent Treatment Advances

**DOI:** 10.3390/medicina57040336

**Published:** 2021-04-01

**Authors:** Guoda Varytė, Daiva Bartkevičienė

**Affiliations:** Clinic of Obstetrics and Gynaecology, Faculty of Medicine, Institute of Clinical Medicine, Vilnius University, LT-03101 Vilnius, Lithuania; daivabartk@gmail.com

**Keywords:** vaginal stenosis, pelvic radiotherapy, vaginal dilation therapy, hyaluronic acid, laser therapy

## Abstract

Radiation-induced vaginal stenosis (VS) is a common side effect of pelvic radiotherapy (RT). RT-induced VS may have various negative effects on women’s quality of life, in particular dyspareunia, decreased vaginal lubrication and difficulties in sexual intercourse. This narrative review provides the aspects of RT-induced VS pathogenesis, incidence, evaluation and associated risk factors. Available treatment modalities are discussed in the article, putting the focus on preliminary, although promising, experience in the use of hyaluronic acid and laser therapy in cancer survivors after pelvic RT.

## 1. Introduction

Treatment of gynecological (vaginal, cervical or uterine) and anorectal cancers commonly requires pelvic radiation therapy (RT). Radiation-induced vaginal stenosis (VS) is a common side effect of pelvic RT and is characterized as an abnormal vaginal canal obstruction due to scar tissue formation [1]. The reported incidence of RT-induced VS varies from 1.2% to 88%, depending on the patient, the type of cancer and treatment (brachytherapy versus pelvic RT, use of dilators, hormones, RT dose, etc.) [2]. According to the latest reports, in the first three years after pelvic RT for endometrial, cervical and anorectal cancer, the estimated rates of VS are respectively 50%, 60% and 80% [3,4,5]. This complication mostly occurs after combined treatment with brachytherapy (BT) and external beam radiotherapy (EBRT). In BT, tissue damage is equally proportioned throughout the whole vagina, whereas in ERBT some areas may be more affected than others. Radiation affects the vaginal epithelium, small vessels and connective tissues, causing local inflammation and cell death. This is followed by vaginal hyalinization and fibrosis due to decreased blood flow, tissue hypoxia, collagen deposition and loss of elastin. Thinned vaginal mucosa, lack of lubrication, processes of scarring and fibrosis leads to a shorter, dryer and less elastic vagina [6]. It is recognized that sexual dysfunction affects more than half of gynecological and anorectal cancer survivors, and at the time of cancer diagnosis some women are young and sexually active [7]. RT-induced VS may have various negative effects on a patient’s quality of life, in particular dyspareunia, decreased vaginal lubrication and difficulties in sexual intercourse. As for the health specialists, RT-induced VS limits gynecological examination during the post-treatment period [8].

## 2. Incidence of Pelvic Radiation Induced Vaginal Stenosis

The described incidence of RT-induced VS varies and depends on the treatment elements, tumor and patient factors. Developing VS is dependent on the site of disease, RT modality and dose, coexisting chemotherapy, and other factors of the patient, such as age or constitutional radio-sensitivity of the tissues. The incidence of RT-induced VS is principally based on small patient groups and retrospective data, also it depends on VS evaluation methods and used grading systems [9]. The reported incidence of RT-induced VS varies from 1.2% to 88% [2]. At this time RT-induced VS is considered a well-known toxicity following pelvic RT. A retrospective study reported a 38% incidence of VS in women treated with pelvic RT and/or vaginal RT for cervical cancer (stages IB to IV). The study reported that mostly VS results in the first year after treatment [10]. According to other studies, VS continuously develops with time, meaning that mild VS occurs in the first year, and moderate or severe VS—three years after the treatment is finished [11]. RT-induced VS in cancer survivors for rectal and anal cancer is less described in the scientific literature. A study conducted by Son et al. reported that 2/3 of patients developed VS following the RT for rectal and anal cancer [12]. Prior studies reported rates of 27.1% (moderate VS) and 37.1% (severe VS) in female patients with anal cancer after chemoradiation treatment [13].

## 3. Pathogenesis

The fast cell turnover of the vulvar and vaginal epithelium supports exfoliation of the outlying layers of non-dividing cells and causes the epithelium to become easily harmed by the radiation treatment. Radiation toxicity is defined by two phases: the acute reaction midst radiotherapy course and late reaction several months after finishing the treatment. In comparison, the late reaction might proceed from the acute reaction or occur after an asymptomatic period of at least three months [14].

VS is a consequence of direct RT injury to the vaginal mucosa. Damage to the small blood vessels and surrounding connective tissues causes decreased blood circulation. This is followed by hypoxia of the vaginal tissues and the progression of telangectasia. Vaginal wall hyalinization and fibrosis forms due to collagen formation in the elastic fibers that compose the vaginal connective tissues. Vaginal atrophy results in a lack of lubrication and thinning of the vaginal mucosa, while decreased vaginal elasticity is due to scarring, circumferential fibrosis and the development of adhesions. For some women, vaginal contractions, narrowing, shortening or total obliteration might occur if vaginal adhesions are not broken down. A reduced or absent ovarian function may lead to estrogen shortage, which further lessens vaginal lubrication and elasticity, intensifies vaginal mucosal thinning and atrophy [15].

## 4. Evaluation of RT-Induced VS

During regular gynecological follow-up visits, detailed information related to vaginal changes is usually picked. However, there is no concise agreement or protocol available for the classification, documentation or grading of radiation induced vaginal injuries and VS. The International Clinical Guideline Group suggested using common terminology criteria for adverse events (CTCAE) and late effects of normal tissues, subjective, objective, management, analytic (LENT SOMA) as measurement tools for defining RT-induced VS However, CTCAE is not specific to RT-induced vaginal injuries (Table 1). The European Organization for the Research and Treatment of Cancer (EORTC) and the US-based Radiation Therapy Oncology Group (RTOG) designed the LENT-SOMA scale. This scale assesses RT side effects, addressing the subjective symptoms of the patient, objective clinical findings, management and analytic investigations (Table 2). The LENT-SOMA grading system demonstrates that symptoms after RT might be an outcome from multiple pathologies and VS is only one integral [2,16,17].

## 5. Associated Risk Factors

The rate and severity of VS is related to the factors of the treatment and the patient. These commonly include age, tumor extension to the vaginal walls and the extent of the vagina treated, radiation dose and a combination of BT with EBRT.

For cervical cancer survivors, there seems to be a direct link between higher radiation dose and vaginal injuries. So far, the widest published study involving 630 women with cervical cancer (from the international study on magnetic resonance imaging (MRI)-guided brachytherapy in locally advanced cervical cancer (EMBRACE) study), identified that the development of VS was associated with a rectovaginal reference point dose. Within 24 months, the estimated risk of developing symptomatic VS was 16% at 55 Gy rectovaginal reference point dose, 20% at 65 Gy, 27% at 75 Gy, 34% at 85 Gy and 43% with 95 Gy [5]. As for uterine cancer and BT, studies showed similar results. A prospective study conducted by Sorbe et al. concluded that vaginal brachytherapy at dose 9.0 Gy per fraction, compared with 6.0, 5.0, or 4.5 Gy per fraction for women with Stage I endometrial cancer was significantly related with shortening of the vagina. Subsequently in five years, shortening of the vagina was 79% against 60%, 50% and 31%, respectively [18]. Further, fifteen years later, the same authors conducted a study of 290 women receiving 2.5 Gy versus 5.0 Gy 6 fractions brachytherapy for early-stage endometrial carcinoma. No significant changes in vaginal length were observed after five years in the 2.5 Gy group, whereas in the 5.0 Gy group, women experienced ¼ vaginal shortening on average [19].

For women diagnosed with endometrial or cervical cancer, it is assumed that a higher volume of vagina treated is associated with an increased risk of VS in the EMBRACE study for cervical cancer—the 24-month actuarial estimate for symptomatic VS was 21%, and the extent of the tumor into the vagina was a considerable risk factor [5]. A few observational studies identified that a longer length of treated vaginal canal significantly increased the risk of VS for women with early-stage endometrial cancer receiving BT [3,20]. There is a lack of studies investigating the effects of RT on vaginal volume and/or portion in cases with pelvic tumors. However, it is known that the lower portion of the vagina has less tolerance to RT compared to the upper or middle portions. Consequently, women have an increased risk of vaginal injuries and VS after lower portion vaginal RT [21].

It is recognized that age >50 years increases the risk of VS for patients receiving pelvic/vaginal RT for cervical cancer [10]. Tobacco has also been identified as a potential patient-related risk factor [14]. This is seemingly possible given the widely known co-active effect of cigarette smoking and radiation reactions on normal tissues of the aero-digestive tract seen in numerous tumors, including cervical tumors [22]. A vaginal pallor reaction is also considered to be a risk factor, knowing that the pathophysiology is associated with atrophy, inflammation, lack of moisture and fibrosis [11].

Within the past few decades, the increased use of RT for vulvar cancer has significantly increased the rates of sexual dysfunction in vulvar cancer survivors. Narrowing of the vaginal introitus, pain and scarring impairs sexual function well before any evidence of vaginal stenosis is present [23,24]. VS in vulvar cancer survivors can be underreported as stenosis of the vaginal introitus creates physical boundaries for gynecological examination and sexual intercourse, therefore, proper vulvar reconstruction surgery is of major importance for these patients [25,26].

## 6. Interventions for Gynecologic and Anorectal Cancer Survivors

### 6.1. Vaginal Dilator Therapy

The idea of vaginal dilator therapy (VDT) after pelvic RT is the early separation and prevention of the adhesions between the walls of the vaginal mucosa. VDT also stretches vaginal tissues and the canal, stimulates epithelial cell growth, preventing circumferential fibrosis and elastosis [27]. Many studies have found significant correlations between VDT and decreased risk of severe VS after pelvic RT for gynecological and anorectal cancers [3,28,29,30,31]. Even though there is no high-level evidence, many reviews and guidelines support the use of VDT after pelvic RT, and dilators remain a generally agreed prevention for VS and are widely used in clinical practice [2,32,33,34].

There are various dilators available from medical supplies shops and many clinics or institutions provide their patients with dilators. Commercially available dilators made of a hard plastic or stainless steel might not be so comfortable for some women to use. Silicone dilators may be used as an alternative and there is a wide assortment of available colors and sizes [35]. As proposed by the International Clinical Guideline Group, women are recommended to start dilation with the smallest size dilator and proceed to larger sizes as VDT comes to be more comfortable. Depending on the cancer site, various shapes of dilators are available. For instance, flat end dilators for uterine and cervical cancer, low end for vaginal and rectal and pointed end for anal cancer. Instructions on how to use dilators may vary according to different clinics, yet the principles are alike. Insertion of the dilator vaginally should be gentle and applied with a water-soluble lubricant. When inserted to the uppermost region of the vagina, the dilator is gently rotated and then removed. VDT should not be administered with physical pressure and injuries to the vagina should be avoided [2].

Unfortunately, there is no specific approach to make VDT uniformly adapted, and recommendations in the scientific literature as to when, how and for how long VDT should be used is inconsistent. The general agreement on many aspects of VDT is lacking, including the regularity and duration of dilator therapy, the optimal VDT initiation time after completing RT, dilator sizes, insertion methods and the need of VDT in sexually active women [2,32,33]. In 2014, a consensus using the Delphi method on VDT for gynecological cancer survivors was reached. It was agreed that radiation oncologists should provide patients with the information about VDT prior treatment. Experts in this field suggested that qualified oncology nurses should provide practical and emotional support. Professionals recommended VDT to prevent vaginal adhesions, shortening and tightening, and it was encouraged to start VDT four weeks after completing RT treatment, perform VDT 2–3 times per week for 1–3 min and to continue VDT for 9 to 12 months. Dilators from a plastic material were recognized as the most suited for VDT [1]. In 2019, the Brazilian version of the consensus reached very much alike conclusions. It recommended VDT should last at least 5–10 min, 2–3 times per week for an indefinite amount of time. However, this consensus did not reach a common agreement on when to start VDT [36]. To date, no existing evidence suggests starting VDT during RT [32].

### 6.2. Hyaluronic Acid Therapy for Vaginal Side Effects after RT

In preventing and neutralizing vaginal injuries after RT for gynecological and anorectal cancers, hyaluronic acid (HA) represents one of the most inventive ways to improve vaginal health in cancer survivors. Local application of HA favors epithelial regeneration, promotes vaginal trophism, elasticity and adequate lubrication. In addition, it sufficiently lessens sticking of the vaginal walls, consequently reducing the development of adhesions, obliteration of the vagina and VS HA is a polysaccharide and part of the of glycosaminoglycans participating in tissue repairment and the regeneration processes. HA is an essential part of the extracellular matrix, which is involved in the repair processes by maintaining sufficient amounts of hydration which assists in the process of cell migration [37]. Due to its high molecular mass, HA is not absorbed when applied on the skin or mucosa. It acts by modeling an invisible, thin and penetrable, visco-elastic outer surface film. This reticular film restores the moisture in the mucosa maintaining the main components of a youthful and healthful tissue, like smoothness, tonicity and elasticity [38]. To this day, HA is extensively used in the clinical practice of various medical branches due to its high level of safety, no contraindications or reports of interactions with other medications [39]. Different vaginal products with HA have been studied with promising results in non-cancer women after menopause [40,41,42].

Several authors investigated the effect of local HA administration and reported reduced treatment related symptoms and vaginal atrophy in patients treated with RT for gynecological cancers. Cassaro et al. examined 45 women with gynecological cancer treated with EBRT with/without vaginal BT. Local treatment with vaginal ovules containing HA reduced dyspareunia, vaginal atrophy, lack of moisture, VS and adhesions [43]. In a study conducted by Dinicola et al., 45 women with cervical cancer treated surgically, with chemotherapy, BT and EBRT received local treatment with HA. Biopsies two months later showed substantially reduced inflammation, fibrosis, cell atypia, bleeding and mucositis [44]. Markowska et al. investigated 37 patients with endometrial cancer after a vaginal BT with complaints of dyspareunia, inflammatory, necrotic lesions and adhesions in the vagina. The patients were administered HA vaginal suppositories, and 86.5% of enrolled patients reported vaginal health improvement after the treatment [45]. In a recent study done by Carter et al. 43 patients with a history of endometrial cancer at least one month after RT (EBRT or vaginal BT) were administered HA containing gel. HL moisturization daily for the first two weeks, and then three times a week until 12–14 weeks significantly improved sexual function and vulvovaginal health [46]. Delia et al. attempted to assess the ability of HA to reduce the side effects of RT for 180 cervical cancer patients. Women in the control group reported the onset and worsening of all symptoms from moderate to severe (inflammation, dryness, dyspareunia), whereas in the group treated with HA suppositories, nearly 90% of women reported none or mild grade symptoms [47]. A study conducted by Laliscia et al. enrolled 126 patients with endometrial cancer who underwent adjuvant vaginal BT and aimed to analyze the effect of HA ovules in the prevention of acute and late vaginal toxicities. The study’s results suggested that patients with intermediate risk endometrial cancer receiving adjuvant vaginal BT after surgery benefited clinically from local HA treatment [48].

### 6.3. Laser Therapy for Vaginal Side Effects after RT

Over the last few years, intravaginal laser therapy showed promising results on sexual health and management of symptoms produced by menopausal genital atrophy. It appears that laser therapy effectively improves sexual function (sexual desire, dyspareunia, vaginal dryness, etc.) and restores vaginal health to a premenopausal state [49,50,51]. Particularly, the CO2 laser is able to activate an acute thermo-ablative or non-ablative effect that stimulates the synthesis of the matured components of collagen, fibroblast proliferation and extracellular matrix that increase vaginal wall elasticity and hydration [52].

There is a lack of studies analyzing vaginal health improvement after laser therapy for patients treated with RT after gynecological and anorectal cancer. Many researchers analyzing intravaginal laser devices in healthy postmenopausal women extrapolate their results to cancer survivors. They propose a theory that most cancer survivors may benefit from intravaginal laser therapy, particularly when local or systemic treatment with estrogen is contraindicated [53]. In a prospective study, Perrone et al. investigated 43 women with severe vaginal shortening, stenosis and atrophy after treatment with EBRT and/or BT for pelvic tumors. Three intravaginal non-ablative CO_2_ laser treatment sessions were scheduled one month apart and all procedures were well tolerated. During the follow up, six months after the last treatment session with the intravaginal laser, examiners observed a progressive increase in vaginal length and Vaginal Health Index (WHI). The authors concluded that intravaginal laser therapy is a promising treatment approach for vaginal injuries for women following pelvic RT, but more randomized clinical trials are needed to confirm the results [54]. A very recent pilot study done by Quick et al. investigated the efficacy of a fractional CO_2_ laser for reducing menopausal genitourinary symptoms in gynecological cancer survivors. The study reported no adverse events and, compared to the placebo, fractional CO_2_ laser therapy demonstrated preliminary evidence of an improvement in sexual function and safety in the gynecological cancer survivors group [55].

### 6.4. Vaginal Estrogen Therapy

Vaginal estrogen is widely used and is recognized as one of the most effective treatment options for menopausal vulvovaginal atrophy. It is proven to be safe in the general non-cancer population [56,57]. Estrogen after RT is thought to promote vaginal epithelium regeneration and the effect may be more evident when vaginal estrogen is administered to the patient three or more months after the RT [14]. It is important to note that vaginal morphological changes caused by radiation have an impact on the effectiveness of vaginal estrogen therapy. A study done by Hofsjö identified that pelvic RT reduces estrogen and androgen protein expression in the vaginal mucosa, demonstrating that women treated with high dose RT have less potential to respond to estrogen treatment [58].

Evidence for the local and systemic hormone therapy management in gynecological cancer survivors is limited and can be challenging considering the risks for disease recurrence and adverse effects. Concerns exist regarding systemic or topical estrogens administered for patients with hormonally responsive tumors or an intact uterus. Based on what is known, estrogen therapy for early-stage endometrial carcinoma (Stages I–II) is reasonable, although no evidence exists supporting hormone therapy in late-stage endometrial carcinoma (Stages III–IV), and as a result, treatment with estrogen is not recommended [59,60,61]. Cervical carcinoma is considered to be a hormonally non-responsive cancer. Ploch et al. conducted a study decades ago investigating 120 women with cervical carcinoma after surgery or RT (Stages I–II). After five years of follow up, the study reported no significant differences in cancer recurrence or survival rates in women receiving hormonal therapy versus the control group [62].

## 7. Conclusions

Radiation induced VS is a well-known side effect following pelvic RT for gynecological and anorectal cancers. Significant improvement in cancer treatment increases the number of cancer survivors who face long-term consequences on their sexual and general quality of life. In the clinical setting, VDT continues to be a globally accepted VS prevention strategy, however, a general agreement on many aspects of VDT is lacking. Although promising, the preliminary experience in the use of hyaluronic acid and laser therapy in cancer survivors after pelvic RT addresses the necessity of sexual rehabilitation for these patients. To confirm the results, further randomized clinical trials comparing and analyzing different treatment options and strategies are mandatory.

## Figures and Tables

**Table 1:** Common terminology criteria for adverse events (CTCAE) for vaginal stricture. Version 5.0.

CTCAE Term.	Grade 1	Grade 2	Grade 3	Grade 4	Grade 5
Vaginal stricture Definition A disorder characterized by a narrowing of the vaginal canal.	Asymptomatic; mild vaginal shortening or narrowing	Vaginal narrowing and/or shortening not interfering with the physical examination	Vaginal narrowing and/or shortening interfering with the use of tampons, sexual activity or physical examination	-	Death

**Table 2:** Radiation Therapy Oncology Group (RTOG)/European Organization for the Research and Treatment of Cancer (EORTC) late effects of normal tissues, subjective, objective, management (LENT-SOMA) scoring table for injury to the vagina.

	Grade 1	Grade 2	Grade 3	Grade 4	Score
Subjective					
Dyspareunia	Occasional and minimal	Intermittent and tolerable	Persistent and intense	Refractory and excruciating	
Dryness	Occasional	Intermittent	Persistent	Refractory	
Bleeding	Occasional	Intermittent	Persistent	Refractory	
Pain	Occasional and minimal	Intermittent and tolerable	Persistent and intense	Refractory and excruciating	
Objective					
Stenosis/length	>2/3 normal length	1/3–2/3 normal length	<1/3 normal length	Obliteration	
Dryness	Asymptomatic	Symptomatic	Secondary dysfunction		
Ulceration/necrosis	Superficial, ≤1 cm^2^	Superficial, >1 cm^2^	Deep ulcer	Fistulae	
Atrophy	Patchy	Confluent	Nonconfluent	Diffuse	
Appearance	Telangiectasia without bleeding	Telangiectasia with gross bleeding			
Synechiae		Partial	Complete		
Bleeding		On contact	Intermittent	Persistent	
Management					
Dyspareunia/Pain	Occasional non-narcotic	Regular non-narcotic	Regular narcotic	Surgical intervention	
Atrophy	Occasional hormone cream	Intermittent hormone cream	Regular hormone cream		
Bleeding	Iron therapy	Occasional transfusion	Frequent transfusions	Surgical intervention	
Stenosis	Occasional dilation	Intermittent dilation	Persistent dilation	Surgical reconstruction	
Dryness	Hormone replacement	Artificial lubrication			
Ulceration	Conservative	Debridement	HBO2	Graft, surgical repair	
Scoring instructions Score the 17 SOM parameters with 1–4. (Score = 0 if there are no toxicities.) Total the scores and divide by 17.					**LENT** **Score:**
Analytic					
Magnetic resonance imaging		Assessment of wall thickness, sinus and fistula formation.			Y/N Date:
Ultrasound		Assessment of wall thickness, sinus and fistula formation.			Y/N Date:
Examination under anesthesia Cytology/biopsy		Assessment of wall diameter and length and mucosal surface.			Y/N Date:
